# Lipoaspirate Shows In Vitro Potential for Wound Healing

**DOI:** 10.3390/pharmaceutics14020447

**Published:** 2022-02-19

**Authors:** Chiara Ceresa, Alessia Borrone, Letizia Fracchia, Maurizio Rinaldi, Alice Marchetti, Carlo Tremolada, Michela Bosetti

**Affiliations:** 1 Dipartimento di Scienze del Farmaco, Università del Piemonte Orientale “A. Avogadro”, 28100 Novara, Italy; chiara.ceresa@uniupo.it (C.C.); a.borrone@hotmail.it (A.B.); letizia.fracchia@uniupo.it (L.F.); maurizio.rinaldi@uniupo.it (M.R.); alice.marchetti@uniupo.it (A.M.); 2 Image Regenerative Clinic, 20122 Milano, Italy; carlo.tremolada@gmail.com

**Keywords:** lipoaspirate, autologous scaffold, MSCs, therapy, tissue regeneration, wound healing, paracrine effect, antibacterial activity

## Abstract

Mesenchymal stem cells (MSCs) are a promising therapy in wound healing, although extensive time and manipulation are necessary for their use. In our previous study on cartilage regeneration, we demonstrated that lipoaspirate acts as a natural scaffold for MSCs and gives rise to their spontaneous outgrowth, together with a paracrine effect on resident cells that overcome the limitations connected to MSC use. In this study, we aimed to investigate in vitro whether the microfragmented adipose tissue (lipoaspirate), obtained with Lipogems^®^ technology, could promote and accelerate wound healing. We showed the ability of resident cells to outgrow from the clusters of lipoaspirate encapsulated in a 3D collagen substrate as capability of repopulating a culture of human skin. Moreover, we demonstrated that the in vitro lipoaspirate paracrine effect on fibroblasts and keratinocytes proliferation, migration, and contraction rate is mediated by the release of trophic/reparative proteins. Finally, an analysis of the paracrine antibacterial effect of lipoaspirate proved its ability to secrete antibacterial factors and its ability to modulate their secretion in culture media based on a bacterial stimulus. The results suggest that lipoaspirate may be a promising approach in wound healing showing in vitro regenerative and antibacterial activities that could improve current therapeutic strategies.

## 1. Introduction

Skin, being the outermost organ that covers the entire surface of the human body, is very common to injury. The loss of intact epidermal and dermal layers can lead to many complications, so prompt treatment of wounds is fundamental to reduce morbidity and mortality. Healing of skin wounds can be challenging, and more than one manner of repair may possibly be fruitful [1].

Traditional wound management options include regular dressings and skin grafting. Skin grafting, which is the transfer of a piece of skin from the donor site to the injured site to close the wound, is the current gold standard of wound treatment even if it creates donor-site morbidity and cannot be used in patients with extensive skin injury [2].

As an alternative, tissue-engineered skin substitutes (TESS) have been developed, but dressing management can often be a slow, time-consuming process because it requires an extended cell expansion period, or in the case of allogeneic TESS that are readily available, can act only as temporary biological dressing [3]. Thus, the need for new therapeutic strategies with higher potential clinical efficacy has fostered the study of stem cells [3,4,5]. Wounds that failed to progress through the normal stages of healing frequently enter a state of pathologic inflammation that is a chronic wound state. Having mesenchymal stem cells (MSCs) immunomodulatory properties, they reduce inflammation by secreting cytokines that promote tissue repair in the lesion. In this way, by attenuating the inflammatory response, they can affect the wound’s ability to progress beyond the inflammatory phase and not to regress to a chronic wound state [6], decreasing fibrosis and therefore reducing scar formation [7]. In addition, MSCs have been shown to tackle microbial pathogens such as *Escherichia coli* and *Staphylococcus aureus* [8], which are one of the factors responsible for a possible wound healing delay and chronicization. Indeed, MSCs produce several antimicrobial peptides, such as LL-37, hepcidin, β-defensin 2, and lipocalin 2, which kill bacteria by the disruption of the membrane integrity, the induction of proinflammatory cytokines release, and the recruitment of immune cells [9,10]. MSCs could be obtained from different anatomical sites [11], but a lot of advantages make adipose tissue the most functional source to be used: it is readily available in large quantities; it can be obtained through low invasive procedures; it gives up to 2% of MSCs (ADSC) compared with only 0.02% in bone marrow (BM-MSC) [12].

MSCs, which are giving promising results for cutaneous wound healing, to date are proposed as cells isolated from the originating tissue, expanded and differentiated in vitro prior to transplantation into the site of damage with or without materials used as scaffolds for cell delivery into the defect area. Due to the extensive time and manipulation necessary to expand sufficient autologous cells, the use of MSC therapy to improve wound healing is limited, as a cell source is not rapidly available after injury.

In our previous study on cartilage repair [13], we demonstrated that these limitations can be overcome, since lipoaspirate acts as a natural scaffold rich in MSCs and gives rise to spontaneous cell outgrowth. Moreover, its paracrine role has been hypothesized, being culture media an inducer of cell proliferation and extracellular matrix (ECM) production [13]. Based on these results, in our present study we hypothesize a similar paracrine and regenerating behavior in other anatomical defects. Hence, we proposed lipoaspirate as an alternative cell-based therapy in wound healing, and we tried to demonstrate whether it could promote and accelerate skin regeneration in minor scars. Adipose-derived stem cells are believed to facilitate healing through differentiation into cells which affect wound healing, e.g., fibroblasts and keratinocytes [14]. They also release pro-healing growth factors and anti-inflammatory cytokines [15] as well as healing-related peptides such as leptin and adiponectin that together may enhance wound healing [16]. Several studies have shown that autologous fat grafting may show significant healing qualities in different clinical situations such as chronically scarred tissue after radiotherapy [17], chronic wounds [18], arterial ulcers [19], pressure ulcers, and diabetic foot ulcers [20].

To determine whether the lipoaspirate can be a useful tool for wound regeneration therapy, we studied in vitro: (1) the ability of resident cells in lipoaspirate to populate a 3D collagen substrate as capability of repopulating a culture of human skin, (2) the effect of lipoaspirate on the proliferation rate and migration of fibroblasts and keratinocytes, (3) the effect of lipoaspirate on fibroblast contraction, (4) the release of trophic/reparative cytokine from lipoaspirate, and (5) the antibacterial effect of lipoaspirate.

## 2. Materials and Methods

### 2.1. Preparation of Lipoaspirate

Lipoaspirate was obtained from five healthy female patients (age range 30–45) undergoing an elective liposuction from a single anatomical site (abdominal subcutaneous fat tissue) at the Image Regenerative Clinic, Milan, Italy. Exclusion criteria were body mass index (BMI) > 30, diabetes, hypertension, and nicotine or alcohol abuse. Preoperative antibiotic prophylaxis was not suggested to the patients, as all the medical procedures were performed in sterility.

Written informed consent, specifying that residual material destined to be disposed of could be used for research, was signed by each participant before the biological materials were removed, in agreement with Rec (2006) 4 of the Committee of Ministers Council of Europe on research on biological materials of human origin.

The skin was previously disinfected with a common antiseptic in the site of liposuction. After a preliminary infiltration of 400 mL of saline solution with 2 mg/mL adrenaline (S.A.L.F. Spa., Bergamo, Italy) as a vasoconstrictor and 0.02% lidocaine (AstraZeneca, Luton, UK) as an anesthetic, the aspiration was performed using a 10-cc syringe with a Luer-Lok^®^ tip (BD Medical, VWR Int., Milano, Italy) connected to a disposable 19-cm blunt cannula (3 mm OD) with five oval holes (1 × 2 mm). No antibiotics were added to the saline solution used for the preliminary infiltration.

From each of five patients, 210 mL of lipoaspirate were processed in the Lipogems^®^ surgery kit [21], and the microfragmented adipose tissue obtained was used in the study and hereinafter referred to as the lipoaspirate. The Lipogems^®^ processing kit is one of the most commonly used procedures to mechanically dissociate adipose tissue. It is a disposable device, filled with a saline solution that reduces the size of the adipose tissue clusters in an “enzyme free” minimal manipulation, through a mild mechanical size reduction using a sequence of sieves and steel beads in a closed and aseptic system. The lipoaspirate clusters generated are a few hundred micrometers in diameter, free from blood and lipids, used with success in multiple indications, spanning cosmetics, orthopedics, proctology, and gynecology [22,23,24]. All the experiments described were performed in parallel at once. When we received the fat from the clinic, half of the lipoaspirate was used to extract hADSCs as described in paragraph 2.4, and the other half was used for the different tests. We never froze the fat or the medium used as lipoaspirate culture medium (Lipo-CM).

### 2.2. Cell Outgrowth from Lipoaspirate

The outgrowth study was done in a 3D matrix as follows: a cluster of lipoaspirate was added to 50 μL of collagen solution, obtained from rat tail tendon [25]. Collagen gel formation was achieved with 1 h of incubation at 37 °C in culture medium (DMEM-F12 with 10% FBS, 100 U/mL penicillin, 100 μg/mL streptomycin, and 2 mM L-glutamine, all from Sigma-Aldrich, Milan, Italy). The kinetics of cell outgrowth and the number of cells/mg tissue were evaluated by quantifying 4′,6-diamidino-2-phenylindole (DAPI; Sigma-Aldrich, Milan, Italy)-positive cells. Briefly, 3D collagen gels were fixed in formalin 4% for 1 h at room temperature and stained for 10 min with 300 nM DAPI (Molecular Probes), before counting nuclear positivity by fluorescence microscopy (Leica Microsystems, Milan, Italy).

### 2.3. Cytokines Detection

The cytokines released by lipoaspirate were assessed using a human cytokine antibody array membrane (Abcam, Cambridge, UK) that detects simultaneously 80 cytokines released from lipoaspirate in culture media or in lipoaspirate lysate. Lipoaspirate (1 mg/mL) was cultured in RPMI added with glutamine and 5% FBS with or without 300 colony forming units (CFU) of *E. coli*. After 24 h incubation at 37 °C in CO_2_, 1 mL culture media was stored at −20 °C before the test, whereas 100 μg lipoaspirate were lysed with 500 μL lysis buffer provided with the kit. After total protein quantification using a Pierce™ BCA Protein Assay Kit (Thermo Scientific, Waltham, MA, USA) according to the manufacturer’s instructions, 1 mg total protein concentration (dilutions done with a buffer supplied by the kit) was incubated on an array membrane containing linked antibodies. Cytokine proteins bind to their antibodies, and then a biotin detection conjugated antibody binds to a second epitope on the protein, creating an antibody "sandwich" around the cytokine. After streptavidin–HRP incubation, membranes were analyzed using a ChemiDoc™ Touch Imaging System (Bio-Rad Laboratories S.r.l., Milano, Italy). A semi-quantitative analysis was performed detecting positive spot density measured by Image Lab™ software (Bio-Rad, Laboratories S.r.l., Milano, Italy). The images were adjusted for positive control normalization and background subtraction and results reported as fold increase compared to negative control.

### 2.4. Cell and Tissue Culture

Normal human fibroblasts (MRC5, ATCC^®^ CLL-171™) were cultured in EMEM (Eagle’s minimum essential medium, EuroClone), NEAA (Lonza, Milan, Italy), 10% FBS, 100 U/mL penicillin, 100 μg/mL streptomycin, and 2 mM L-glutamine (all from Sigma-Aldrich, Milan, Italy).

Human keratinocytes (HaCaT; ATCC, Manassas, VA, USA) were cultured in DMEM (Sigma-Aldrich, Milan, Italy) supplemented with 1% L-glutamine and antibiotics (Sigma-Aldrich, Milan, Italy), and 10% FBS (EuroClone).

Human adipose-derived stem cells (hADSCs) were isolated as stromal cell fraction from lipoaspirate with digestion with type I collagenase 1 mg/mL in PBS (Sigma-Aldrich, Milan, Italy) at 37 °C for 30–60 min, centrifugation for 10 min at 450× *g*, pellet resuspension in blood cell lysis buffer (2.06 g/L Tris base, pH 7.2, and 7.49 g/L NH_4_Cl; Sigma-Aldrich, Milan, Italy), and incubation at room temperature for 10 min. Pellets were collected and filtered sequentially through 100- and 40-μm cell strainers (VWR Int.) to remove undigested tissue. The pellets were then washed with PBS and the cells resuspended in DMEM/Ham’s F12 medium (*v*/*v* 1:1) supplemented with 40% FBS, 100 U/mL penicillin, 100 μg/mL streptomycin, and 2 mM L-glutamine.

Lipoaspirate was cultured in RPMI with 5% FBS, supplemented with 2 mM L-glutamine. The lipoaspirate culture media (LipoCM) was used to test cytokines and to test its activity on proliferation, migration, and contractibility of MRC5 and HaCaT. In detail, LipoCM was obtained as follows: lipoaspirate (1 mL) corresponding to 850 ± 20 mg of tissue was cultured in 10 mL of basal medium in a CO_2_ incubator at 5% CO_2_, 37 °C for 4 days.

### 2.5. Cell Proliferation Assay

MRC5 and HaCaT were plated in 48-well plates in 500 uL of LipoCM at a density of 2.5 × 10^3^ for fibroblasts and 5 × 10^3^ for keratinocytes. Proliferation was tested using an ATP quantification kit (ViaLight, Cambrex Profarmaco, Milan, Italy). The manufacturer’s protocol was followed, and all reagents used were supplied with the kit; briefly, cells were lysed with cell lysis buffer and treated with ATP monitoring reagent, which utilizes luciferase. The light produced was measured by a luminometer (Victor X4; PerkinElmer, Milan, Italy) and expressed as relative luminescence units (RLUs). For fibroblasts (MRC5) with LipoCM, RLUs were measured at 0, 24, 48, and 72 h of culture. For keratinocytes (HaCaT), RLUs were measured at 0, 72, and 144 h of culture. A basal control (cells in culture media without any added treatment) and a positive proliferation control (cells supplemented with 10 ng/mL FGF2) were included. Results were expressed as proliferation ratio with respect to time 0. The experiment was carried out in duplicate and repeated three times (*n* = 6).

### 2.6. Scratch Wound Healing Assay

For the migration activity of lipoaspirate on fibroblasts and keratinocytes, 1.5 × 10^5^ cells were plated in 24-well plates in 500 μL of medium and incubated at 37 °C in 5% CO_2_ until confluence and then serum starved for 20–24 h. The monolayers were wounded by the introduction of two linear perpendicular scratches, creating a cross with a sterile pipette tip, rinsed with PBS, cultured in a medium containing 2% FBS without or with the LipoCM, and compared with a positive control (hyaluronic acid 200 µg/mL) and a negative control (DMSO 3%). The fields (30–40 µm) were selected at a fixed distance from the border of the culture plate. To evaluate the migration, the cells were observed using a Leica digital camera connected to an inverted microscope (all from Leica Microsystems, Milan, Italy). For fibroblasts (MRC5), images were acquired at 0, 6, 12, and 24 h. For keratinocytes (HaCaT), images were acquired at 0, 12, 24, and 48 h. The distances crossed by the cells were measured using the Qwin Image Analysis system (Leica Microsystems, Milan, Italy). Results were expressed as migration ratio with respect to time 0. The experiment was carried out in triplicate and repeated three times (*n* = 36).

### 2.7. Collagen Gel Contraction Assay

To determinate the effect of lipoaspirate on matrix contraction, a collagen gel (3.5 mg/mL) was obtained from rat tail tendons by acetic acid extraction [25]. Fifty microliters of collagen were added to 50 μL of fibroblasts (1 × 10^5^ cells/mL) in serum-free basal media prior to gel formation, and the solution was allowed to polymerize for 30–45 min at 37 °C. The solidified gels were gently detached from the plastic surface to allow contraction. The gels were then incubated at 37 °C in 5% CO_2_ in 1 mL of serum-deprived medium treated with or without the tested stimuli:

Blank: collagen with culture medium (without cells and stimuli);
Ctr: collagen with cells without stimuli;
Ctr+: collagen with cells + TGFβ1 5 ng/mL;
LIPO: collagen with cells + 15% *v/v* of LipoCM.

To determine the extent of matrix contraction, the matrices were measured at 0, 24, 48, 72, and 144 h. Images of the gels were taken, and measures of areas were done using an inverted microscope equipped with a digital camera connected to an image analysis system (all from Leica Microsystems). Contraction data were obtained as the change in diameter after 0, 24, 48, 72, and 144 h and expressed as area reduction ratio with respect to time 0. The experiment was carried out in triplicate and repeated three times (*n* = 9).

### 2.8. Antibacterial Effect of Lipoaspirate

The antibacterial activity of lipoaspirate and its conditioned medium (CM) was evaluated as indicated in Krasnodembskaya et al. [26]. *Escherichia coli* ATCC 25922 and *Pseudomonas aeruginosa* ATCC 10145 were used for these experiments. The strains were stored at −80 °C in tryptic soy broth (Biolife Italiana, Monza, Italy) supplemented with 25% glycerol, and, for each experiment, they were grown overnight at 37 °C on tryptic soy agar plates.

The inhibition of *E. coli* growth by lipoaspirate was assessed in 12-well plates and compared to that observed with lipoaspirate-derived hADSCs. Briefly, lipoaspirate (1.00 ± 0.04 g) or hADSCs (2 × 10^5^ cells/well) in 1 mL of RPMI with 5% FBS + 2 mM L-glutamine were co-incubated with 300 CFU at 37 °C for 6 h at 5% CO_2_. Positive (*E. coli*) and negative (lipoaspirate and hADSCs w/o bacteria) controls of microbial growth were also included.

The antibacterial activity of lipoaspirate and hADSCs conditioned medium (CM) was tested in 96-well plates. Briefly, the CMs of lipoaspirate or hADSCs, pre-incubated with or w/o *E. coli* for 6 h, were collected, centrifuged (10 min at 15,000 rpm), and stored overnight at −20 °C. After thawing, aliquots of CM (90 μL) or fresh media were inoculated with 100 CFU of *E. coli* and *P. aeruginosa* (in 10 μL of PBS) and incubated for 16 h at 37 °C. At the end of the incubation time, aliquots of culture medium were serially diluted with PBS, and each dilution was plated in triplicate onto McConkey agar for *E. coli* count or cetrimide agar for *P. aeruginosa* count (Scharlab, Barcelona, Spain) in triplicate. CFU were counted after overnight incubation at 37 °C. The experiment was carried out in triplicate and repeated three times (*n* = 9).

### 2.9. LL-37 Quantification

A commercial enzyme-linked immunosorbent assay (ELISA) kit was used for LL-37 peptide measurement (Hycult Biotechnology, Vinci-Biochem, Italy) according to the manufacturer’s instructions. The LL-37 concentration in culture media of lipoaspirate (0.5 g/mL) or hADSCs (5 × 10^5^ cells/mL) co-incubated with or w/o 300 CFU *E. coli* for 6 h and 24 h was quantified. Briefly, standards and samples (50 μL) were aliquoted to the pre-coated plates in duplicate. After 1 h of incubation and washing, 50 μL of conjugate were added. After 1 h of incubation and washing, 100 μL of enzyme substrate were added to each well and incubated for 15 min before adding 100 μL of solution. The optical density of each micro-well was read using a microplate reader at 450 nm, and the level of LL-37 in each well was calculated using a logarithmic standard curve (requiring an R2 value over 95%); the average of the duplicates was used as the results. The data obtained were reported as percentage of the LL-37 increase in lipoaspirate and hADSCs culture medium after stimulation with *E. coli*.

### 2.10. Statistical Analysis

All analyses and graphics were performed using the statistical program R, 3.6.2. (R Core Team (2019). R: A language and environment for statistical computing. R Foundation for Statistical Computing, Vienna, Austria. URL https://www.R-project.org/, accessed date 13 January 2022). To estimate log10 CFU/mL from colony counts, the R package dupiR was used [27]. The value of *p* < 0.05 was considered to reflect statistical significance.

## 3. Results

### 3.1. Cell Outgrowth from Lipoaspirate and Secretory Activity

**Figure 1:**
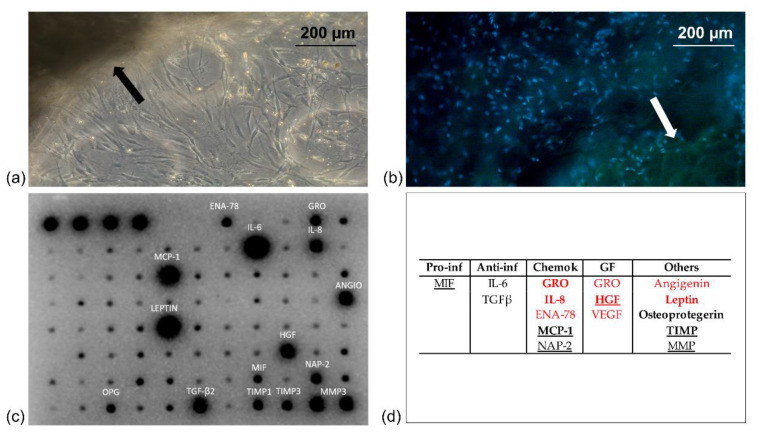
Lipoaspirate clusters, indicated with an arrow in the microscopic images, cultured in basal medium: (**a**) phase-contrast microscopy of cell outgrowth in a 3D collagen matrix; (**b**) fluorescence microscopy of DAPI stained cells outgrown from lipoaspirate cluster; (**c**) protein detected in supernatants, classified in (**d**) according to their activity. Underlined are those with known activity in wound healing, in bold those with antimicrobial activity, in red those with angiogenic activity.

Figure 1a,b reports representative images of a lipoaspirate cluster cultured in a 3D collagen showing the outgrowth of cells after 3 days of culture. Both images, taken with a Leica digital camera connected to an inverted microscope (Figure 1a) or to a fluorescence microscope (Figure 1b DAPI staining), showed clearly the ability of cells to outgrow lipoaspirate.

Collagenase digestion used to extract hADSCs reduced the secretory activity of stromal vascular cells when compared to the cultured lipoaspirate clusters (BCA protein quantification, result not shown) confirming the results obtained by Vezzani et al. that have demonstrated this effect both qualitatively and quantitatively [28]. The lipoaspirate releases cytokines produced by the different cell populations that are part of it such as adipocytes, pericytes, mesenchymal stem cells, endothelial cells, fibroblasts, macrophages, and T lymphocytes. Analyzing the secretome of lipoaspirate, we found in the culture media a high secretion of growth factors that embrace angiogenic, chemotactic, growth, and antimicrobial roles (Figure 1c). The several cytokines found were classified according to their activities in five groups: proinflammatory (Pro-inf), anti-inflammatory (Anti-inf), chemokines (Chemok), growth factors (GF), and others, as shown in Figure 1d.

Among them, vascular endothelial growth factor (VEGF), angiogenin, ENA-78, GRO, IL-8, leptin, hepatocyte growth factor (HGF), and TIMP could be considered as growth factors with angiogenic activity. In addition, some of them are chemotactic cytokines that have regulatory functions both in cell growth and in the inflammatory response, such as GRO, ENA-78, NAP2, and IL-8. In culture media, we also found a high secretion of factors that are known to be involved in wound healing and tissue regeneration, such as MCP-1, MIF, MMP, TGF-β2, and NAP2, and others that reduce inflammation, such as OPG and IL-6. Moreover, other factors detected have a direct or indirect antimicrobial role, such as IL-6, ENA-78, GM-CSF, GRO, GROα, IL-6, IL-8, IL-10, MCP-1, MCP-2, MCP-3, MIG, MIP-1β, RANTES, angiogenin, leptin, GPC-2, HGF, IP-10, LIF, MIF, MIP-3α, osteoprotegerin, PIGF, TGFβ2, TIMP-1, and TIMP-2.

We have observed that some proinflammatory factors such as TNF-α or IL-1β were detected only at low levels, whereas a higher quantity of MIF was observed. Anti-inflammatory factors and chemokines were represented, respectively, by IL-6, IL-8, TGFβ ENA-78, NAP-2, and macrophage inflammatory protein (MIP)-1β. Vascular endothelial growth factor (VEGF), hepatocyte growth factor (HGF), and GRO were growth factors detected together with other factors including high levels of leptin, angiogenin, matrix metalloproteinase (MMP)-3, and inhibitors (TIMP).

### 3.2. Effect of Lipoaspirate on Fibroblasts and Keratinocytes Activity

**Figure 2:**
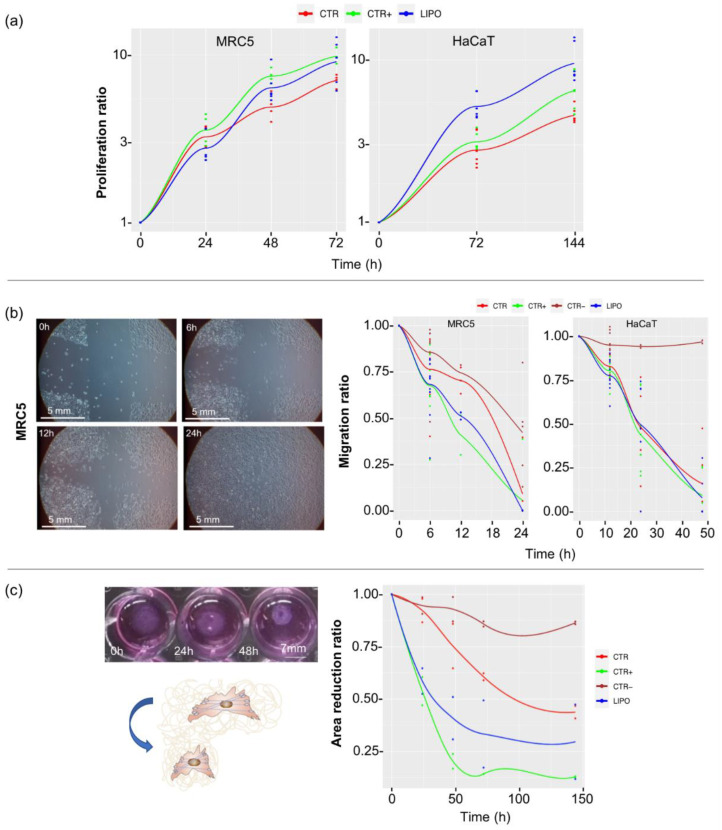
Proliferation, migration, and contraction activity of lipoaspirate: (**a**) proliferation data obtained by ATP via light assay kit at 0, 24, 48, and 72 h for fibroblasts (MRC5) and 0, 72, and 144 h for keratinocytes (HaCaT). Results are expressed as proliferation ratio with respect to time 0; (**b**) scratch wound healing assay image of MRC5 (image in LIPO condition taken from time 0 to time 48 h). A cross scratch has been created on a confluent monolayer of MRC5 and HaCaT cells. The width of the branches was measured at different times: for fibroblasts at 0, 6, 12, and 24 h, and for keratinocytes at 0, 12, 24, and 48 h. Line graph reports the cells migration ratio with respect to time zero. (**c**) Contraction of a collagen gel with fibroblasts at 0, 24, 48, 72, and 144 h compared to time 0. The figure shows the area decrease of the gel during time. Results are expressed as area reduction ratio with respect to time 0. CTR = basal media; CTR+ = basal media added with FGF2 (10 ng/mL) in proliferation and migration tests, added with TGFbeta1 (5 ng/mL) in the contraction test; CTR− = DMSO 3% in migration test, LIPO = basal media added with 15% *v/v* of LipoCM.

In Figure 2, the results of proliferation (Figure 2a), migration (Figure 2b), and contraction (Figure 2c) of cells are reported. Proliferation test data are expressed as proliferation ratio with respect to time 0. ATP quantification showed an almost double increase in proliferation of keratinocytes (HaCaT) treated with LipoCM (LIPO) compared to control keratinocytes (CTR) both at 72 h and at 144 h (*p* < 0.01). Moreover, the LipoCM showed higher proliferative activity compared to keratinocytes treated with FGF2 used as known positive proliferative stimulus (CTR+) [29]. Fibroblasts (MRC5) also increased in their proliferation when treated with LipoCM (LIPO) compared to the basal proliferation control (CTR).

To investigate the LipoCM migration induction on skin resident cells, we reproduced a wound condition in vitro creating two perpendicular scratches in a confluent cell monolayer. The closure of the scratch was mediated by cell migration, and the width of the cross branches was measured at different times. Data were expressed as the migration ratio with respect to time 0. FGF2 was chosen as a positive stimulus according to its known action on fibroblast migration [30]. Fibroblasts showed a statistically significant migratory increase of the LipoCM condition (LIPO) compared to control at 12 h of incubation (*p* < 0.05) with migration ratio values that did not differ significantly from the ratio values of the CTR+. After 24 h of culture, all the results were comparable due to the closure of the gap in all the experimental conditions, including control, so this time point was not useful in our evaluations. When analyzing data of keratinocyte (HaCaT) migration, no differences between the stimuli used were observed, but only from the basal control at 24 and 48 h.

Contraction data shown in Figure 2c, expressed as contraction ratio with respect to time 0, were obtained measuring the collagen gel area during time. Results evidenced a 50% of area contraction of CTR at 144 h. In comparison to CTR, LIPO demonstrated a higher contraction power (*p* < 0.05), and this difference was shown for all the experimental times. Comparing the LIPO with CTR+ (TGFβ1), no statistical differences were observed at 24 h with 50% of contraction induced; at further times set (48, 72, and 144 h), CTR+ contraction was significantly higher than the LIPO, evidencing more that 80% of contraction instead of the 65% of the LIPO.

### 3.3. Antibacterial Effect of Lipoaspirate and Its Conditioned Medium

To simulate an infection condition and study if lipoaspirate could modify its cytokine pattern and its antibacterial activity, lipoaspirate was co-cultured with 300 colony forming units (CFU) of *E. coli*. Data were compared to those obtained from the same experiment done on isolated hADSC.

**Figure 3:**
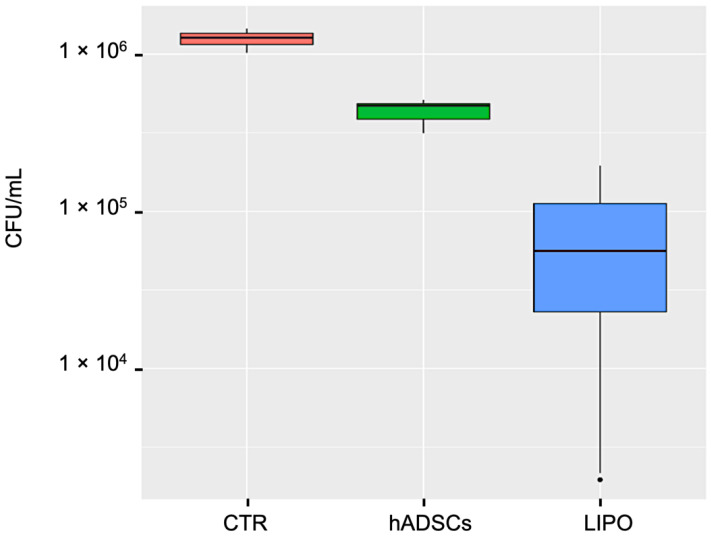
Lipoaspirate and hADSCs antibacterial activity against *E. coli*. Bacterial growth was assessed by the viable cell counting method and expressed as CFU/mL. CTR = positive control of *E. coli* growth: basal media inoculated with 300 CFU of *E. coli*; LIPO = lipoaspirate in basal media inoculated with 300 CFU of *E. coli*; hADSCs = lipoaspirate derived hADSCs in basal media inoculated with 300 CFU of *E. coli*.

The antibacterial activity of lipoaspirate (LIPO) and lipoaspirate derived hADSCs after 6 h of co-incubation with *E. coli* is shown in Figure 3. The results obtained were analyzed by one-way ANOVA followed by Tuckey post-test. In general, compared with the positive control (CTR), the growth of *E. coli* was significantly reduced by LIPO (*p* < 0.001) and hADSCs (*p* < 0.001), with percentages of inhibition of 95% and 67%, respectively. In particular, LIPO showed a marked and more pronounced antibacterial effect (+28%) in comparison to hADSCs (*p* < 0.001).

**Figure 4:**
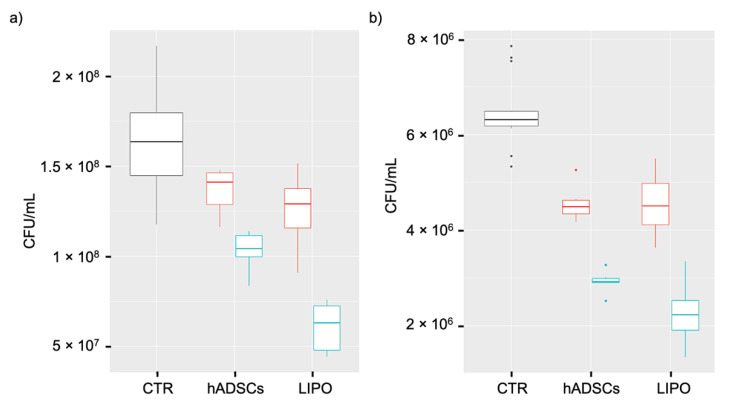
Antibacterial activity of lipoaspirate and hADSCs conditioned medium against *E. coli* (**a**) and *P. aeruginosa* (**b**). Bacterial growth was assessed by the viable cell counting method and expressed as CFU/mL. CTR = positive control of bacterial growth: basal media inoculated with 100 CFU of bacterial strain; LIPO = lipoaspirate conditioned medium inoculated with 100 CFU of bacterial strain; hADSCs = lipoaspirate derived hADSCs conditioned medium inoculated with 100 CFU of bacterial strain; – = not previously co-incubated with *E. coli*; + = previously co-incubated with *E. coli*.

The antibacterial activity of LIPO and hADSCs conditioned medium (CM), previously co-incubated with (+) or w/o (–) *E. coli*, against *E. coli* and *P. aeruginosa*, is shown in Figure 4. One-way ANOVA followed by Tuckey post-test was used to analyze the differences among CTR and CM w/o pre-incubation with *E. coli* (hADSCs– and LIPO–). Two-way ANOVA followed by Tuckey post-test was used to analyze the differences among CM w/o pre-incubation with *E. coli* (hADSCs– and LIPO–) and CM previously co-incubated with *E. coli* (hADSCs+ and LIPO+). Compared to CTR, the growth of *E. coli* and *P. aeruginosa* was significantly reduced by LIPO– (*p* < 0.001) and hADSCs– (*p* < 0.01). In the case of *E. coli*, inhibitions of 23% (LIPO–) and 17% (hADSCs–) were detected, whereas for *P. aeruginosa*, a reduction of bacterial growth of 30% (LIPO–) and 29% (hADSCs–) was found. No significant differences were observed between hADSCs– and LIPO– (*p* > 0.05). Furthermore, for both the bacterial strains, stimulated samples showed a marked and more pronounced antibacterial effect in comparison to the corresponding conditioned media without prior bacterial stimulation (*p* < 0.001). In particular, for *E. coli*, stimulation increased the antibacterial activity of hADSCs and LIPO of +20% and +41%, respectively. For *P. aeruginosa*, stimulation increased the antibacterial activity of hADSCs and LIPO of +26% and +35%, respectively. In addition, LIPO+ was more effective in comparison to hADSCs+ (*p* < 0.05), with an increase of inhibitory activity of +27% (*E. coli*) and +10% (*P. aeruginosa*).

### 3.4. Cytokines Profile Modification after E. coli Infection of Lipoaspirate

**Figure 5:**
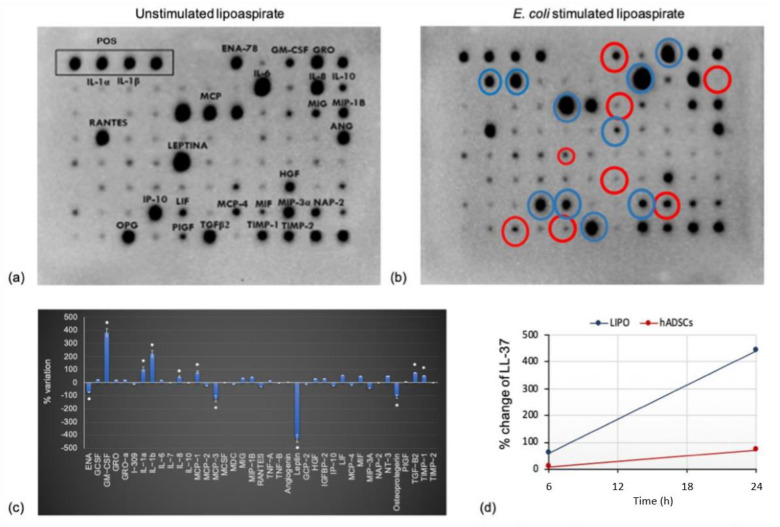
Cytokine release of unstimulated lipoaspirate (**a**) and cytokine release of *E. coli* stimulated lipoaspirate (**b**) after 24 h coincubation. Blue: cytokines with increased level, red: cytokines with lowered levels. Semiquantitative analysis expressed as % variation of cytokines profile after *E. coli* stimulation (**c**). Change of LL-37 amount in lipoaspirate culture medium (LIPO) and hADSCs culture medium (hADSCs) after 6 h and 24 h of co-culture with 300 CFU/mL of *E. coli* compared with unstimulated samples (**d**).

Cultured lipoaspirate proved to be viable and active, as demonstrated by its ability to modify the pattern of cytokines released, when stimulated with LPS used as a pro-inflammatory factor (result shown in a previous paper Sabbatini et al. [31]) or with bacteria, as shown here in Figure 5. When the cytokine pattern of *E. coli* infected LIPO (Figure 5b) was compared to that of the untreated control (Figure 5a), considered the basal secretory activity, a significant increase of release for the GM-CSF (granulocyte monocyte colony stimulating factor), IL-1α, IL-1β, MCP-1, TGFβ 2, and TIMP-1 cytokines was observed, whereas a reduction in the release of osteoprotegerin (OPG) and leptin was detected. As shown in Figure 1, the lipoaspirate already contained some cytokines at the basal level, produced by the different cells that constitute it. These cytokines are ENA-78, GM-CSF, GRO, GROα, IL-6, IL-8, IL-10, MCP-1, MCP-2, MCP-3, MIG, MIP-1b, RANTES, angiogenin, leptin, GPC-2, HGF, IP-10, LIF, MIF, MIP-3α, osteoprotegerin, and PIGF, TGFβ2, TIMP-1, and TIMP-2. The intensity of the spot is an approximate release level indicator. Some of them increased after the bacterial stimulation (round circle in blue in Figure 5b), while others decreased (round circle in red), and still others appeared of constant intensity. LIPO + chemiluminescence results were elaborated and reported in Figure 5c, confirming more precisely what was observed at chemiluminescence. Cytokines, whose production appeared constant, underwent a slight increase, as in the case of GRO and GRO-α. A significant increase in production/secretion was observed for the cytokines GM-CSF (granulocyte monocyte colony stimulating factor), IL-1α IL-1β, MCP-1, TGFβ 2, and TIMP-1; while OPG (osteoprotegerin), leptin, ENA-78, and MCP-3 were released in lower amounts. An increased release was observed for the GM-CSF (granulocyte monocyte colony stimulating factor), IL-1α, IL-1β, MCP-1, TGFβ 2, and TIMP-1 cytokines, whereas a reduction in the release of osteoprotegerin (OPG) and leptin was detected.

### 3.5. LL-37 Quantification

Lipoaspirate and hADSCs secreted the antimicrobial molecule LL-37. An increase in LL-37 release was observed in the culture media of lipoaspirate and hADSCs due to *E. coli* stimulation, as shown in Figure 5d.

The quantification of LL-37 in the medium of unstimulated hADSCs and LIPO demonstrated that LL-37 is present at baseline and its concentration increases following a bacterial stimulus, as clearly observed already after 6 h of infection, with a high increase after 24 h. The graph shows, in blue, the increased levels of LL-37 after the bacterial stimulus in lipoaspirate cultures and in orange the increased levels of LL-37 after the bacterial stimulus in hADSCs cultures. The increases were calculated as ratio of the difference in LL-37 amount between the samples with or without bacterial stimulation and the baseline LL-37 level; hADSCs stimulated with 300 CFU/mL showed, after incubation, an increase of LL-37 from a basal level of 19.27 ng/mL to 33.03 ng/mL, while the LIPO, placed in the same experimental conditions, showed an increase from a basal quantity of 2.35 ng/mL to 12.67 ng/mL. These data showed that the increase of LL-37 level from untreated to *E. coli* treated cultures was much higher in LIPO culture than in extracted hADSCs, even if ADSCs produced higher quantities of LL37 than LIPO.

## 4. Discussion

Skin lesions represent an ongoing challenge in therapies that address wound care, and as the number of patients increases, technology aimed at stimulating wound healing becomes inefficient. Research on the impaired healing process is proceeding hastily as evidenced by new therapeutic approaches other than conventional, such as single growth factor, dual growth factors, skin substitutes, cytokine stimulators, cytokine inhibitors, matrix metalloproteinase inhibitors, gene and stem cell therapy, and extracellular matrix and angiogenesis stimulators. Regenerative medicine has emerged as an alternative therapeutic option to improve wound healing and restore normal skin architecture [32], in addition to the therapeutic use of platelet-rich plasma (PRP) and its derivatives [33] and stem cell-based therapy, which has become a promising new approach in the field of regenerative medicine [34]. In each of these approaches, the role of growth factors in the complex and overlapping phases of wound healing has been studied consistently over time [35,36,37]. It has been suggested that there is a deficit of growth factors in chronic wounds and that the use of exogenous growth factors brings benefits to the healing process.

Adipose tissue contains mesenchymal cells and is easily extracted with a liposuction procedure from the abdominal and/or gluteal region in local anesthesia, allowing to obtain a viable autologous scaffold rich in stem cells with a minimally invasive cheap and fast intervention. In particular, its direct use instead of isolated cells, would limit the excessive manipulation to isolate stem cells, reduce the costs deriving from the isolation, expansion, and implantation of the cells and avoid the use of synthetic or natural scaffolds, which could create problems of biocompatibility and poor integration. It is also established that non-isolated mesenchymal cells keep longer vitality and functionality without going into senescence, and therefore their use in the lesion site in their natural habitat could lead to greater effectiveness [38]. The microanatomy of lipoaspirate clusters obtained with Lipogems^®^ technology is the same as adipose tissue in 0.4 ± 0.1 mm area but free from oil and blood residuals. In a loose connective tissue, protein secretion resulted not only from hADSCs but contained also factors secreted by the other cells that constitute the cluster, such as preadipocytes and mature adipocytes with capillaries between them, pericytes wrapped around endothelial cells, and leukocytes.

It has been shown that mesenchymal cells, contained in high amounts in lipoaspirate, secrete positive paracrine substances for the migration of fibroblasts within the tissue of granulation of the wound [39]. We hypothesized and then demonstrated that a similar behavior can be obtained by directly using the lipoaspirate containing the stem cells, without excluding that other residing cells (preadipocytes, pericytes, blood cells, endothelial cells, dendritic cells) could be responsible or co-responsible for active paracrine secretions. The lipoaspirate, during the 10 days of culture, releases various growth factors and cytokines active on cellular functions that could be responsible for the functional modifications seen on eukaryotic and prokaryotic cells.

The presence of these factors in the lipoaspirate supports our idea that the cytokines and chemokines released into the culture medium by the different cell populations may display an antibacterial activity by a direct or indirect action on cells involved in the immune response. From such evidence, it can be inferred that the clinical setting in which a direct application of lipoaspirate is proposed may not be limited only to wound healing.

Concerning the antibacterial activity, we first demonstrated a direct effect of both lipoaspirate and lipoaspirate-derived hADSCs against *E. coli* cells co-incubated for 6 h. The activity of hADSCs against bacterial pathogens is well known; Krasnodembskaya et al. [26] proved that human bone marrow-derived MSCs were able to inhibit *E. coli* growth directly, after 6 h co-incubation. In addition, they demonstrated that their conditioned media were significantly active against *E. coli* and *P. aeruginosa*, with growth inhibition percentages of 40% and 70%, respectively, and the antimicrobial activity was ascribed to the secretion of the antimicrobial peptide, human cathelicidin hCAP-18/LL-37.

Interestingly, in our study, the lipoaspirate itself was significantly more active (+28%) than the lipoaspirate-derived hADSCs against *E. coli* with an inhibition percentage of 95%, confirming our hypothesis that other cell populations might have taken part in the antibacterial activity both acting directly or indirectly.

In order to study whether lipoaspirate could modify its antimicrobial activity in the presence of a bacterial stimulus, we compared the conditioned media of lipoaspirate and hADSC previously co-incubated or not with *E. coli*. In addition to the increased antibacterial effect against *E. coli* and *P. aeruginosa* of both conditioned media obtained after bacterial stimulation, we demonstrated, for the first time, that the conditioned medium of stimulated lipoaspirate was significantly more effective than that of stimulated hADSCs, both against *E. coli* and *P. aeruginosa.* This substantiates the idea that other residing cells aside for hADSCs could be responsible or co-responsible for active paracrine secretions, and that the use of the whole lipoaspirate in wound healing could bring multiple benefits. To understand this result, it has to be considered that LL37 was produced by 5 × 10^5^ ADSCs, whereas for LIPO culture, we can only suppose a stem cell number that could be 10 times lower, because we cultured 0.5 g of tissue, and from literature, we know that in 1g there are about 10^5^ stem cells [11]. Additionally, in this case and as shown by other studies [40,41], collagenase digestion used to extract hADSCs reduced the secretory activity of stromal vascular cells, moreover when stimulated with an external factor, e.g., bacteria in our experiments, isolated cells responded extremely less to the stimulus.

Furthermore, the present study allowed us to highlight the in vitro cellular response of fibroblasts and keratinocytes to lipoaspirate, assuming its autologous topical use for the promotion of skin wound healing. The effect on skin cells was tested using the medium that had come into contact with lipoaspirate and not the lipoaspirate in co-culture, since it was observed that when co-cultured, lipoaspirate was able to release cells autonomously (Figure 1a) that were indistinguishable from fibroblasts and keratinocytes, thus interfering with the experimental results. The data of proliferation, migration, and contraction of cells obtained in vitro in this work could therefore be underestimated, since they derived from the activation mediated by factors released by the resident cells in lipoaspirate, but in vivo the cellular response could also be influenced by the cells migrating from the lipoaspirate.

Not much is known about how bacterial pathogens get in contact and interact with the cells of the lipoaspirate. A study by Fiedler et al. [42] investigated the impact of wound infection by relevant Gram-positive and Gram-negative bacteria and their cell wall components (lipopolysaccharide and lipoteichoic acid) on human adipose tissue-derived mesenchymal stem cells (ADSC). They demonstrated that the tested species were able to attach to and internalize into ADSC, that bacterial antigens had an effect on ADSC proliferation and differentiation, and that ADSC-controlled regeneration was not necessarily decreased under infectious conditions.

In our study, the LPS of the external membrane of the *E. coli* cell wall could have acted as a trigger for the release of trophic/reparative cytokine and for the increased antibacterial activity of lipoaspirate. Increased paracrine effects and enhanced regenerative and repair features have been observed in LPS-preconditioned mesenchymal stromal cells [43], and some studies have demonstrated that LPS exposure of MSCs can enhance their trophic effects and functional properties to defend against the harsh inflammatory environment [44,45].

Lipoaspirate ability to release into the culture medium factors that stimulate the regeneration of the cells populating the dermal and epidermal layer is supported by other recent studies [46,47,48]. In addition, the medium that was in contact with the lipoaspirate showed mitogenic activity for fibroblasts in addition to stimulating its contraction in a short time. Fibroblasts indeed, under the stimulus of different growth factors (PDGF, TGFβ, EGF, and FGF), can differentiate into myo-fibroblasts expressing higher quantity of α smooth muscle actine (α-SM actine) that allows contractility. This activity is fundamental during the wound healing process; fibroblasts actively contract, bringing closer the two gap sides, thus promoting wound closure. Whether this contraction is lacking, the healing could be delayed with possible chronic development or fibrotic scar formation.

Moreover, we demonstrated that, together with an intrinsic secretory activity, cultured lipoaspirate responds to bacteria co-culture increasing the secretion of antimicrobial factors, thus explaining the antimicrobial activity observed in our study.

Many of the cytokines produced and released by lipoaspirate, and shown in the dot blots of Figure 1 and Figure 5, have already demonstrated an anti-inflammatory role [28], and some of them are also involved in tissue repair processes. The in vitro antibacterial activity of the lipoaspirate observed in our study, however, requires an investigation on the potential role that these cytokines may play in the antimicrobial response as well as the understanding if some of them, released in greater quantities after stimulation, and/or already present at basal level, have a direct or indirect inhibitory activity on bacterial growth. The presence of interleukin 8 (IL-8) in the lipoaspirate is an advantage in favor of its use as an adjuvant in wound healing, due to its anti-inflammatory, tissue repair, and antibacterial properties of this cytokine. IL-8 is a chemokine secreted by several cells of inflammation and from epithelial cells that is not just a chemo-attractor factor for monocytes and neutrophils, but also stimulates the secretion of NADPH-oxidase by phagocytes [49]. Its antibacterial activity has been assumed to reside in the portion generated by its cleavage, as for other peptides belonging to the cathelicidin family, such as LL-37 and GROα.

HGF is a cytokine released by several cells that are part of the lipoaspirate, such as fibroblasts and mesenchymal cells. Its binding to the Met receptor causes several biological responses, including re-epithelialization and tissue regeneration; stimulation of motility and migration of endothelial cells, melanocytes, and keratinocytes; and involvement in the processes of angiogenesis and wound repair. Among the different downstream responses of the HGFMet complex, there is also the immune response modulation, having a chemo-attractor effect on the neutrophils and an inhibitory role against chronic inflammation and fibrosis [50,51].

Among the cationic cytokines, MCP-1 was found, together with MCP-2, at a basal level in the lipoaspirate culture and was released in higher amounts after bacterial stimulation. Having in vitro [52] and in vivo [53,54] bactericidal activity, this cytokine may be indicated as responsible for the antibacterial activity observed in the microbiological tests. In addition, it demonstrated wound healing properties, showing activities on re-epithelialization, angiogenesis, and synthesis of collagen.

TIMP-1 is a cytokine found at the basal level in the lipoaspirate, which increases after stimulation (Figure 5). It belongs to a family of four members called metallo-protease inhibitors. TIMP-1 and TIMP-2 are able to inhibit metallo-proteases that play a central role in the repair and tissue remodeling, controlling the balance between ECM deposition and its own degradation in various physiological and pathological processes [55]. As for the antibacterial activity, a correlation between TIMP-1 and the metalloproteases was detected in vivo, showing resistance to *P. aeruginosa* in TIMP-1-deficient mice [56].

The cell population with the greatest phagocytic activity is that of macrophages, which are activated by several cytokines, including GM-CSF [57], which is released in the epidermis during the wound healing process, increasing the proliferation of keratinocytes and the consequent re-epithelialization together with its angiogenic role. It affects the proliferation, chemotaxis, and cytotoxic properties of macrophages, recalls neutrophils during damage, and promotes their action at the injured site. Being one of the cytokine with the most marked increase in release from lipoaspirate following contact with bacteria (Figure 5b,c), we can assume that its effects on the production and number of circulating neutrophils, together with its modulation of numerous actions of mature cells (adhesion, movement, phagocytosis, oxidative stress, secretion, and degradation), can increase the microbicidal capacity [58].

In our study, following microbial stimulation, a significant increase of other cytokines with important biological roles, including IL-1, was observed. LPS stimulates mononuclear phagocytes to produce IL-1 in vitro, which may act indirectly by stimulating migration and activity of phagocytic cells that, increasing the production of proteins in the acute phase, seem to support the bactericidal activity of macrophages. This allows a more rapid and efficient activation of T lymphocytes and the increase of interferon-γ release, which is necessary for the activation of macrophages [59]. Furthermore, IL-1 induces the expression of FGF-7 by fibroblasts promoting indirect re-epithelialization, and promotes the synthesis of metalloproteases (MMPs) together with inhibiting the synthesis of ECM and TIMPs.

A significant increase in bacterial stimulated lipoaspirate is also observed for transforming growth factor (TGFβ-2). In vitro, its pro-mitogenic activity on fibroblasts has been demonstrated together with its inhibitory activity towards keratinocytes, so its role may appear paradoxical, and therefore it is considered a negative regulator of re-epithelialization in wound healing [60]. However, it induces the expression of integrins necessary for the migration of keratinocytes and for their anchoring to the matrix. In several animal models, the administration of exogenous TGF is important to speed up wound healing, by stimulating angiogenesis, the proliferation of fibroblasts, the differentiation of myofibroblasts, and matrix deposition [61]. The role of TGFβ2 in tissue remodeling and repair is certainly relevant, but the same cannot be said for the antibacterial activity. The fact that LIPO + releases this cytokine in greater quantities, however, confirms the dynamic role of this tissue in the wound healing process, where the cooperation of all the cytokines produced is required.

Two proteins, OPG and leptin, produced at high levels in lipoaspirate decreased significantly when lipoaspirate was co-cultured with *E. coli*. The OPG belongs to the superfamily of TNF receptors and is known to be involved in bone resorption, but its role in wound healing and as an antimicrobial has to be studied and understood, considering its high level observed in the basal tissue culture (LipoCM).

Leptin, physiologically produced by adipocytes [62,63], has been shown to be advantageous both in vitro and in vivo in murine models of wound healing, in particular by acting on keratinocytes and thus favoring the re-epithelialization. In addition, in local infection sites, where the tissue contains several adipocytes that produce leptin, phagocytic activity appears up-regulated, and neutrophils are stimulated by leptin to secrete reactive oxygen species. Interestingly, in our study, leptin is the cytokine that undergoes one of the most drastic reductions following contact with *E. coli* in vitro. This decrease might be explained by the fact that *E. coli* can exploit this cytokine as a nourishment for its survival and self-maintenance during the 24 h co-culture with the lipoaspirate.

The fact that LL-37 is released by hADSCs confirms that the mesenchymal cells isolated not only from bone marrow but also from adipose tissue are able to release LL-37 into the culture medium [64], as also evidenced in our study. The release of LL-37 into the lipoaspirate culture medium is the result of a similar behavior of the intact tissue with respect to the cells isolated. Furthermore, higher levels of LL-37 are found in the lipoaspirate culture medium compared to the hADSC medium, reinforcing our idea of achieving a greater effect with the direct use of lipoaspirate in/on the injured site, not only as an anti-inflammatory and regenerative agent, but also as an antibacterial. hMSCs are in fact known to be beneficial in treating infections associated with both Gram-negative and Gram-positive pathogens by releasing the antimicrobial peptide LL-37 [64]. In our study, we demonstrated an increase in LL-37 release in the conditioned medium of lipoaspirate and hADSCs after *E. coli* stimulation compared to the non-stimulated, which was reflected in a significantly higher antibacterial effect against both *E. coli* and *P. aeruginosa* in stimulated samples. In addition, stimulated lipoaspirate was more effective than stimulated hADSCs against both pathogens.

Based on the results obtained, the lipoaspirate can thus be considered as a valid alternative to hADSC, even superior to them, limiting the difficulties and obstacles related to their isolation. The hADSCs LL-37 value measured by the ELISA test is higher than that obtained in the reference publication [26] that was carried out on mesenchymal cells deriving from bone marrow. This difference could result from a different production/release capacity of LL-37 from stem cells from different anatomical sites and/or from different stimulation times with bacteria, which in our case are 24 h against the 6 h reported in the literature.

The data obtained in this study turn out to be promising and could be the basis for future in vivo tests. However, several limitations and technical issues have to be taken into account and addressed to avoid biases. Individual variables must be considered; in fact, each lipoaspirate obtained has different characteristics based on the anatomical site of sampling, age, gender, and pathologies of the patient. In addition, the method used for lipoaspirate treatment can also affect its ability to release cells and active factors into the microenvironment in which it will be used.

## 5. Conclusions

The ability of resident cells in lipoaspirate to grow out without enzymatic digestion and the paracrine effect on the proliferation rate, migration, and contraction of fibroblasts and keratinocytes gives lipoaspirate a great appeal for chronic wound repair. These results together with the release of trophic/reparative cytokine and the high antibacterial role of lipoaspirate could constitute a promising adjuvant to antibiotic therapy, determining an effective therapeutic advantage for the healing of wounds in the field of regenerative medicine.
